# Reduced‐Dimensional Reaction‐Space Analysis of a Trispericyclic Reaction by ReSPer

**DOI:** 10.1002/jcc.70539

**Published:** 2026-11-02

**Authors:** Takuro Tsutsumi, Akifumi Fujita, Yuriko Ono, Tetsuya Taketsugu

**Affiliations:** ^1^ Department of Chemistry, Faculty of Science Hokkaido University Sapporo Japan; ^2^ Institute for Quantum Chemical Exploration Tokyo Japan; ^3^ Institute for Chemical Reaction Design and Discovery (WPI‐ICReDD), Hokkaido University Sapporo Japan

**Keywords:** dimensionality reduction, dynamical branching reaction, pericyclic reaction, post‐transition‐state bifurcation, potential energy landscape

## Abstract

Post‐transition‐state bifurcation, which plays a critical role in determining product selectivity, has gained significant attention in synthetic chemistry. A trispericyclic reaction has been reported, producing three different cycloadducts from a single transition state structure. Its mechanisms have been examined through elementary reaction processes and dynamical trajectory simulations. However, because the relevant static reaction paths and dynamical trajectories are embedded in a high‐dimensional configurational space, their mutual relationships and the potential‐energy features governing product branching are difficult to interpret without choosing a limited set of reaction coordinates. In this study, we use the Reaction Space Projector (ReSPer) method, which mathematically constructs a reduced‐dimensional chemical reaction space from a set of reference structures to uncover the dynamical branching mechanisms of the trispericyclic reaction. The two‐dimensional reaction space reveals that the reaction path network in the configurational space forms an arrowhead‐shaped landscape, differing from the traditionally recognized fork‐shaped landscape. Projecting dynamical trajectories onto the two‐dimensional reaction space shows that dynamic branching processes occur near the shoulder in the energy profile along the main reaction path from the transition state to the major cycloadduct, where the potential valley orthogonal to the reaction direction flattens out. These results demonstrate that ReSPer provides a useful reduced‐dimensional representation for relating static reaction‐path networks to dynamical branching trajectories, and may offer new descriptors for future branching‐ratio prediction in pericyclic reactions.

## Graphical abstract

Reduced‐dimensional reaction‐space analysis reveals that a trispericyclic reaction follows an arrowhead‐shaped potential‐energy landscape rather than a simple fork‐shaped bifurcation. Projection of AIMD trajectories onto this space clarifies how rare IRC‐jump events from a shoulder region lead to minor products.

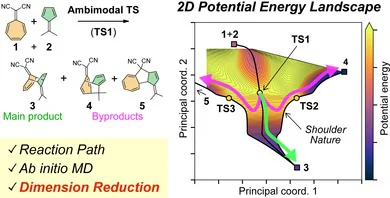

## 1 Introduction

Post‐transition‐state bifurcation (PTSB) refers to the phenomenon where a single transition state (TS) leads to two or more products (Figure 1) [1, 2, 3]. This process has been observed in a variety of chemical reactions, including organic synthesis [4, 5, 6, 7, 8, 9, 10], transition metal‐catalyzed reactions [11, 12, 13], and biosynthesis [14, 15, 16]. In 2002, Caramella et al. identified the bispericyclic TS, where two TSs connecting common reactants to different cycloadducts converge into a single TS. Houk et al. then introduced the term “ambimodal TS” for pericyclic reactions resulting in multiple cycloadducts [17] and reported various bis‐, tris‐, and tetra‐pericyclic reactions [18, 19, 20]. Numerous researchers have since worked on predicting and controlling the selectivity of PTSB reactions, developing practical prediction models focusing on critical structures within the configurational space [21, 22, 23, 24, 25, 26]. These key structures include the ambimodal TS, the second TS, product A, and product B, as shown in Figure 1.

**FIGURE 1:**
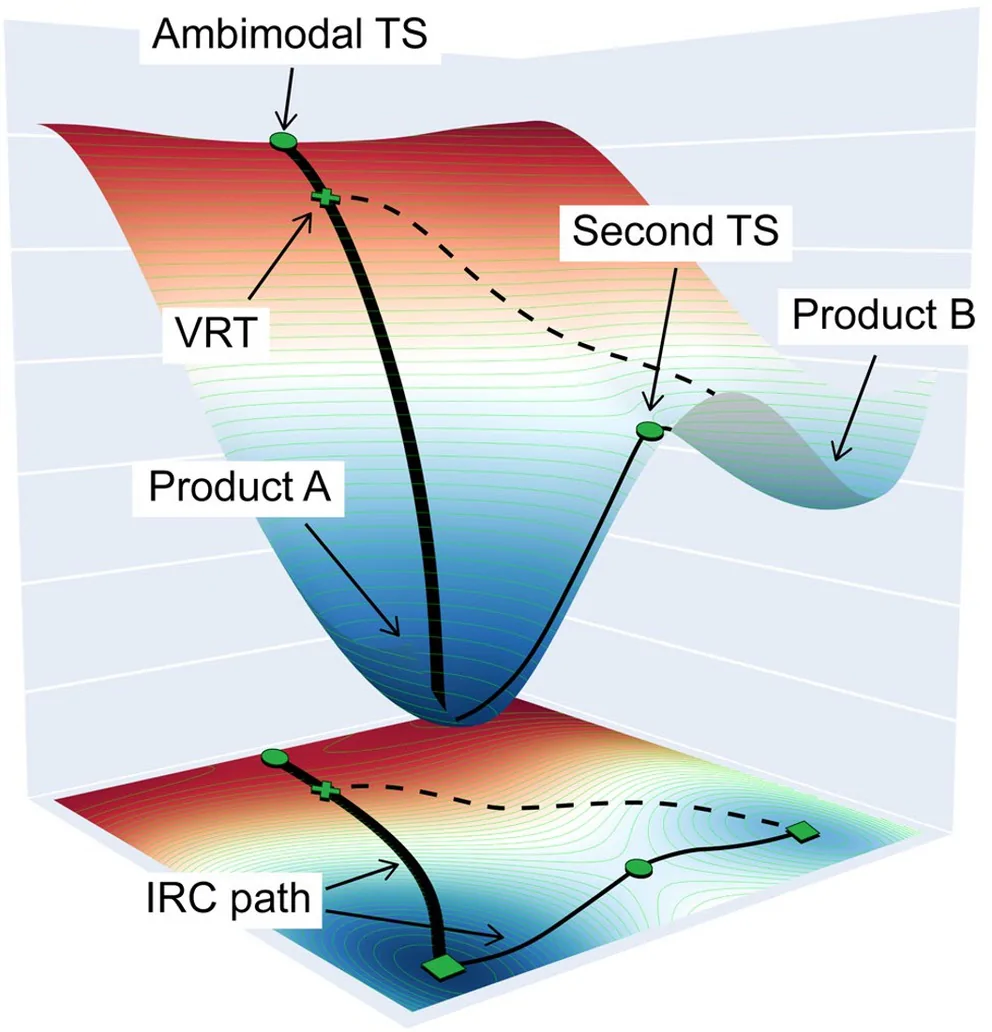
Two‐dimensional model surface for a post‐transition‐state bifurcation (PTSB) reaction showing how a single ambimodal TS structure can lead to two different products via a valley‐ridge transition (VRT) point along the intrinsic reaction coordinate (IRC) path.

The selectivity of branching reactions is influenced by the shape of the potential energy surface (PES). The intrinsic reaction coordinate (IRC) is the path of steepest descent starting from a TS and connecting a reactant to a product structure on the PES [27, 28, 29]. For an *N* atomic molecule, the IRC path descends the valley floor in the 3*N*‐6 dimensional PES. According to the IRC picture, the static reaction path diverges into multiple valleys at the valley‐ridge transition (VRT) point. This is where the valley floor suddenly changes to a ridge line concerning the reaction coordinate [30, 31, 32]. The IRC calculation cannot identify reaction path bifurcations, except in cases where it terminates at the second TS, because the IRC path only follows the tangent vector of the potential gradient [33]. Over the past two decades, exhaustive reaction path search methods [34, 35, 36], such as the anharmonic downward distribution following (ADDF) [37] and the artificial force induced reaction (AFIR) [38], have been developed. The relationships between the analytical pathways defined by ADDF [39] and AFIR [40] and reaction path bifurcations have been studied [41].

Ab initio molecular dynamics (AIMD) is a classical trajectory method that uses atomic forces calculated via quantum chemical methods to provide atomic‐level reaction behavior on the full‐dimensional PES. Such a trajectory descends the PES, winding around the IRC valley, and can jump to another IRC path regardless of the presence of a VRT. We refer to these dynamical behaviors as IRC‐jumps [42]. Therefore, AIMD simulations have been employed to study the selectivity and mechanisms of branching reactions [4, 5, 6, 7, 8, 9, 10, 12, 13, 14, 15, 18, 20, 26].

Several research groups have thoroughly revealed the mechanisms and driving forces of PTSB reactions by analyzing IRC paths and classical trajectories based on model PES and low‐dimensional subspaces [1, 2, 3, 4, 5, 6, 7, 8, 9, 11, 12, 13, 14, 15, 17, 18, 19, 20]. Traditionally, this subspace is defined by selecting important internal coordinates. However, choosing reaction coordinates is not unique and heavily depends on chemical intuition, leading to some arbitrariness. Even if one can identify reaction coordinates representing multiple processes involved in the target reactions, the complexity of chemical reactions can quickly make the number of coordinates exceed human‐visible dimensions. Previous studies have constructed a two‐dimensional subspace for bifurcation reactions by referring the forward IRC path from the ambimodal TS to the second TS and the complete IRC path connecting two different products via the second TS [43]. However, describing multi‐branching reactions with three or more different products remains challenging due to human visibility constraints. Recently, Houk and co‐workers reported several pericyclic reactions leading to three and four different cycloadducts [18, 20], highlighting the need for a practical, general method to uniquely construct low‐dimensional PES for multi‐branching reactions.

We have developed the Reaction Space Projector (ReSPer) method to uniquely determine principal coordinates (PCos) representing the positional relationship of molecular structures in full‐dimensional space and project them into a reduced‐dimensional subspace [44, 45, 46, 47]. The ReSPer method has been extended to project classical trajectories into the low‐dimensional reaction space defined by two or three PCos, revealing dynamical reaction processes. From these studies, we found that the IRC‐jump process drives branching reactions alongside the conventional process traversing the VRT [46, 47]. The ReSPer analysis has recently been extended to excited‐state dynamics, such as the photochemical reactions of *cis*‐stilbene [48] and *o*‐nitrophenol [49]. Several researchers have also constructed low‐dimensional reaction spaces using a dimensionality‐reduction method other than ReSPer and discussed PTSB mechanisms based on trajectories projected into the two‐dimensional space [9, 10].

Although previous IRC and trajectory studies have provided important insights into pericyclic reactions with PTSB processes, the relationship between the static IRC network and the ensemble of dynamical trajectories has remained difficult to visualize in a unified configurational space. This difficulty becomes more serious for multi‐branching reactions that lead to three or more products, where the conventional fork‐shaped schematic picture may not fully represent the actual arrangement of reaction paths on the high‐dimensional PES.

Here, we clarify the mechanism of the trispericyclic reaction (Scheme 1) [18] based on the reduced‐dimensional reaction space constructed using ReSPer. We also examine the dynamical PTSB processes by projecting AIMD trajectories initiated from the ambimodal TS onto the predefined two‐dimensional reaction space. The procedure of this study is as follows. First, referring to the previous study, [18] IRC calculations are performed from the ambimodal TS and related Cope rearrangement TSs connecting different cycloadducts. Second, a two‐dimensional reaction space is constructed from the dominant PCos obtained by ReSPer, and an approximate potential energy landscape around the projected IRC network is visualized in this space. Finally, AIMD trajectories starting from the ambimodal TS are projected onto the two‐dimensional reaction space to clarify the dynamical behavior during the trifurcating PTSB processes.

**SCHEME 1:**
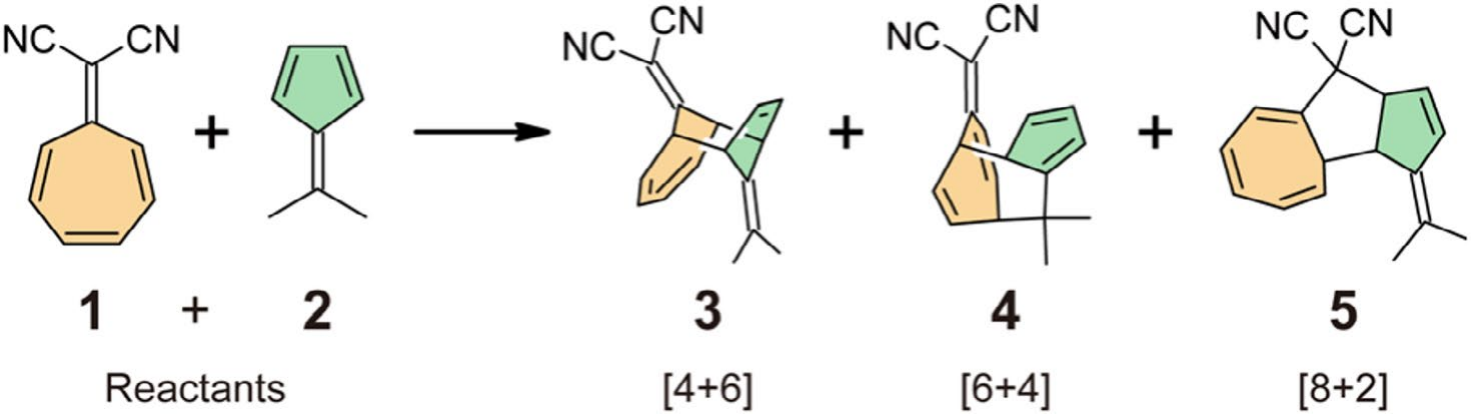
Trispericyclic reaction of 8,8‐dicyanoheptafulvene (**1**) and 6,6‐ dimethylfulvene (**2**) leading to [4 + 6] (**3**), [6 + 4] (**4**), and [8 + 2]‐cycloadducts (**5**), proposed by Houk et al. [18].

## 2 Computational Details

### 2.1 ReSPer

ReSPer is a robust tool for constructing a reduced‐dimensional reaction space using reference structures from IRC paths and analyzing dynamical reaction behavior by projecting AIMD trajectories into this low‐dimensional space [44, 45, 46]. By applying ReSPer to any given set of reference structures, we can determine PCos that closely reproduce the relationships of structural distances in the high‐dimensional configuration space. This allows us to extract the low‐dimensional subspace defined by two or three PCos without the ambiguity associated with selecting reaction coordinates.

The ReSPer scheme consists of four steps: (1) prepare a dissimilarity (Euclidean distance) matrix **D** from a reference structure dataset, which serves as input data for a dimensionality reduction method; (2) perform dimensionality reduction to obtain PCos; (3) construct a reduced‐dimensional reaction space using the dominant PCos; and (4) if necessary, project AIMD trajectories onto the predefined reaction space. In this study, we selected portions of related IRC paths as the reference structure dataset. For the dimensionality reduction method, we employed classical multidimensional scaling and its out‐of‐sample extension technique. The Euclidean distance *d*_*ij*_ between molecular structures *i* and *j* was calculated as follows:

(1) $${\left(D\right)}_{\mathrm{ij}}=d_{\mathrm{ij}}=\left|\xi^{\left(i\right)}-\xi^{\left(j\right)}\right|,$$

where $\xi^{\left(i\right)}$ is the mass‐weighted Cartesian coordinate of structure *i*. Before the distance calculation, the two structures were optimally overlapped using the Kabsch algorithm [50]. More detailed descriptions can be found in the literature [46, 47].

### 2.2 Electronic Structure Calculations and AIMD Simulations

The geometry optimization and reaction path searches were conducted using the AFIR method [38]. The Cartesian coordinates of all products and TS structures discussed in this article are provided in Section 5 of the Supporting Information. We also simulated AIMD trajectories at the same computational level using the SPPR program [51]. The time evolution of Newton's equation of motion was conducted by the velocity‐Verlet algorithm. Initial conditions were determined by normal‐mode sampling at the ambimodal TS: atomic coordinates and velocities were generated by adding 0.5 *k*_B_ *T* (*T* = 298 K) to the reaction mode and 1.5 *hν* (the energy of the first excited state of a harmonic oscillator) to the other vibrational modes, in accordance with the law of equipartition of energy. The time step was set to 0.4 fs, and 200 trajectories were propagated toward the product region until typical bond lengths, shown in Figure 2a, satisfied the following conditions: (i) bond‐a and bond‐b, (ii) bond‐a and bond‐c, and (iii) bond‐a and bond‐d are all 1.6 Å or less; or (iv) bond‐a, bond‐b, bond‐c, and bond‐d are all 4.0 Å or more. These conditions correspond to the formation of each cycloadduct and the absence of a reaction. All electronic structure calculations were performed using density functional theory (DFT) with the ωB97XD functional and the 6‐31G(d) basis set, implemented in the Gaussian16 program [52]. The ωB97XD/6‐31G(d) level of theory was used to maintain consistency with the previous computational study of the same trispericyclic reaction by Houk and co‐workers [18]. This level of theory provides a practical balance between computational cost and accuracy for IRC and AIMD simulations, while including empirical dispersion correction through the ωB97XD functional.

**FIGURE 2:**
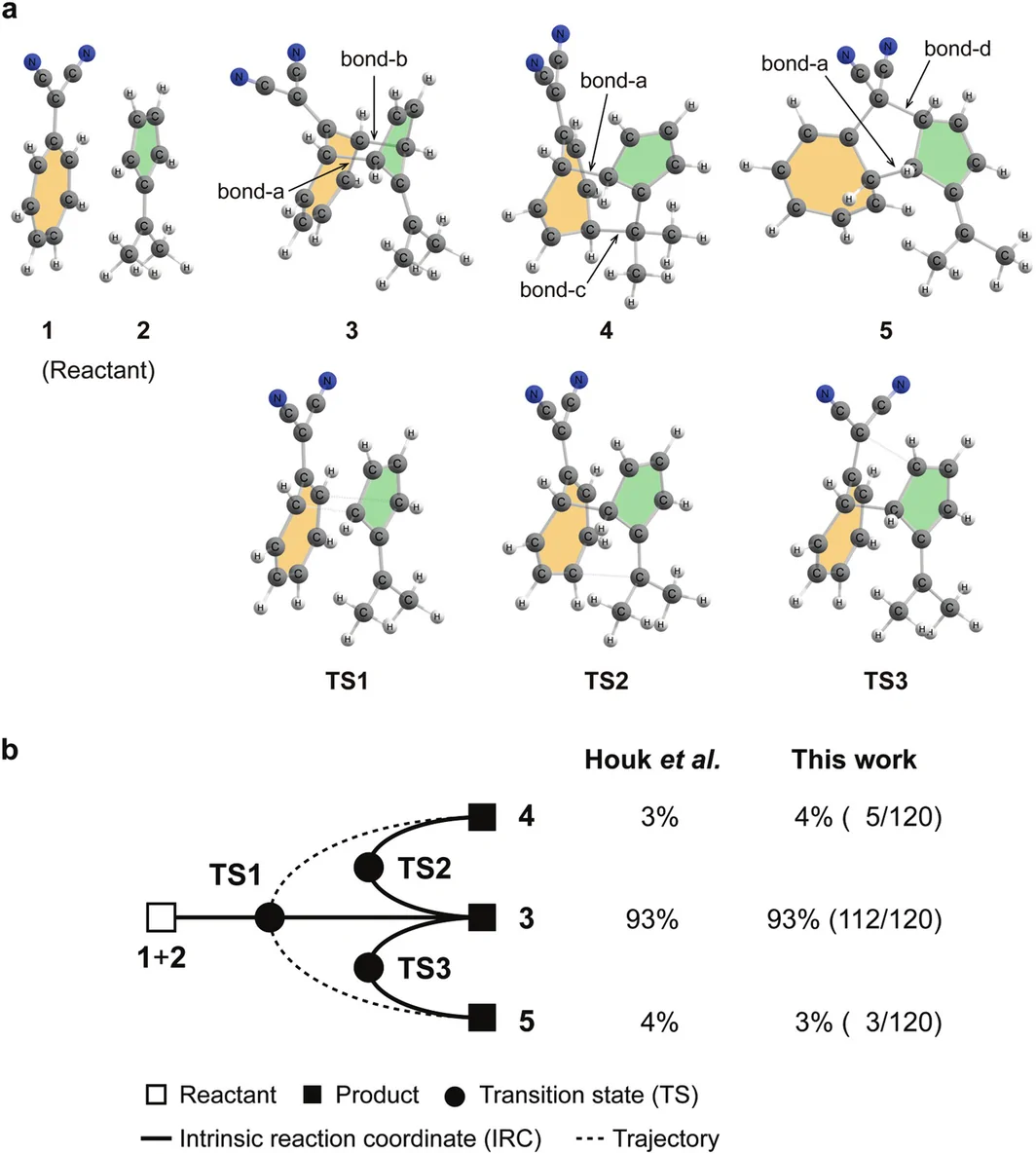
Key molecular structures and a schematic illustration of the trispericyclic reaction. (a) Molecular structures of the reactants (**1** + **2**), the [4 + 6]‐cycloadduct (**3**), the [6 + 4]‐cycloadduct (**4**), the [8 + 2]‐cycloadduct (**5**), TS1 connecting **1** + **2** and **3**, TS2 connecting **3** and **4**, and TS3 connecting **3** and **5**, all optimized at the ωB97XD/6‐31G(d) level of theory. (b) Schematic illustration of the post‐transition‐state trifurcation along the IRC, along with the branching ratios observed in both the previous [18] and current studies.

## 3 Results and Discussion

### 3.1 Reaction Paths and Branching Ratio

Referring to the previous study [18], we performed reaction path exploration and the AIMD simulations. Figure 2a shows the optimized geometries of the reactants, three cycloadducts, and three TS structures. TS1 connects the reactants (**1** + **2**) with the [4 + 6]‐cycloadduct (**3**), TS2 connects **3** with the [6 + 4]‐cycloadduct (**4**), and TS3 connects **3** with the [8 + 2]‐cycloadduct (**5**). The key C–C bond lengths of these optimized structures are summarized in Table S1 (Section 1 of the Supporting Information). Notably, TS1 corresponds to the ambimodal TS, while TS2 and TS3 correspond to the Cope rearrangement reactions. Other reaction paths connecting **1** + **2** with **4** or **5** could not be obtained using the double‐sphere (DS‐) AFIR method, which finds a single path linking two given structures [53]. Following the previous study by Houk and co‐workers [18], we simulated 200 trajectories initiated at TS1. Of these, 80 trajectories returned to the reactant region, while the remaining 120 trajectories reached **3**, **4**, and **5** in the proportions of 112, 5, and 3 trajectories, respectively. The resulting branching ratio is **3**:**4**:**5** = 93:4:3, which is quantitatively consistent with the previously reported ratio of **3**:**4**:**5** = 93:3:4 [18], supporting the characterization of TS1 as an ambimodal trispericyclic TS leading to three different cycloadducts. A schematic illustration of the present post‐transition‐state trifurcation reactions is summarized in Figure 2b.

### 3.2 Reaction Path Analyses

We first examined the frequency variation of the vibrational modes orthogonal to the IRC along the IRC connecting **1** + **2** and **3** to confirm the existence of a VRT point that triggers the trifurcating reaction of interest. Figure 3a shows the low vibrational frequencies along the IRC from TS1 to **3**, which are perpendicular to the reaction coordinate, revealing a VRT point at *s* = 4.01. However, as shown in Figure 3b, the direction of the imaginary frequency (125.7*i* cm^−1^) at the VRT promotes a branching reaction related to methyl‐group rotation rather than C–C bond formation. Figure 3a also indicates that the shape of the PES perpendicular to the IRC becomes shallower at *s* = 0.98 and 3.19; however, the corresponding structural changes for the lowest frequencies are unrelated to C–C bond formation. Additionally, we examined the electronic density changes induced by structural changes along the three IRCs using the natural reaction orbital method [54, 55], as depicted in Figure S1 (Section 2 of the Supporting Information).

**FIGURE 3:**
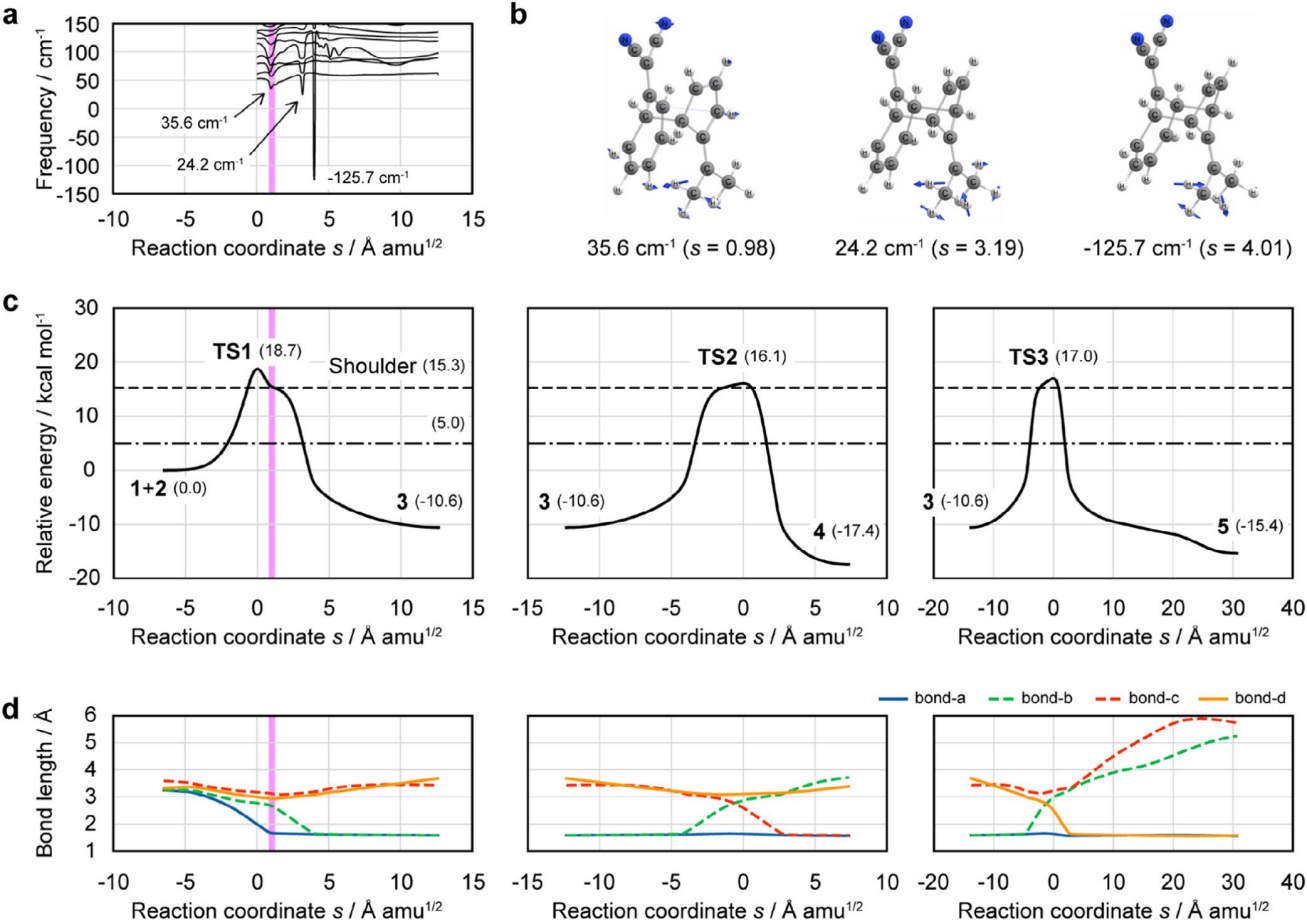
Vibrational analysis, potential energy profile, and significant bond lengths along the IRCs starting from TS1, TS2, and TS3. (a) Vibrational analysis along the forward path of IRC1. (b) Molecular structures and vibrational modes corresponding to the local minima of the lowest frequencies in (a). (c) Relative energy profiles along IRC1, IRC2, and IRC3. The dashed and dash‐dotted lines represent the potential energy of the shoulder region of IRC1 and the 5 kcal/mol threshold, respectively. (d) Variations in the four C–C bond lengths shown in Figure 2a, with bond‐a, bond‐b, bond‐c, and bond‐d represented by blue lines, green dash lines, red dash lines, and orange lines, respectively.

To further characterize the shape of the potential energy landscape, we analyzed the potential energy profiles and the four C–C bond lengths along three IRCs. The IRC paths originating from TS1, TS2, and TS3 are referred to as IRC1, IRC2, and IRC3, respectively. Figure 3c shows the potential energy profiles along these three IRCs. Figure 3d illustrates the changes in the four C–C bond lengths: bond‐a is common to all cycloadducts, while bond‐b, bond‐c, and bond‐d are crucial to the formation of **3**, **4**, and **5**, respectively. Interestingly, the energy profile along IRC1 from the TS1 to **3** exhibits a shoulder, whereas those along IRC2 and IRC3 feature plateaus. The relative energies at TS2 and TS3 are only 0.8 and 1.7 kcal/mol above the shoulder of IRC1. At this shoulder, the bond lengths of bond‐c and bond‐d, corresponding to cycloadducts **4** and **5**, simultaneously shorten. These characteristics of the potential energy landscape suggest that the shoulder of IRC1 plays a pivotal role in the entrance to the three reaction channels leading to **3**, **4**, and **5** [56]. Additionally, the potential valley perpendicular to IRC1 becomes shallower at the shoulder, as shown in Figure 3a. Therefore, we predict that trajectories originating from TS1 diverge into three reaction channels leading to **3**, **4**, and **5** from the IRC1 shoulder via IRC‐jump processes, where a trajectory dynamically crosses over an energy barrier between two IRCs.

### 3.3 Reduced‐Dimensional Reaction Space Constructed by ReSPer

To uncover the dynamical branching processes based on the potential energy landscape, we constructed a reduced‐dimensional reaction space using the ReSPer program. Based on our preliminary analysis (Figure S2 and Section 3 of the Supporting Information), we selected 71 structures with relative energies of 5 kcal/mol or higher along the three IRCs as reference structures and constructed a two‐dimensional reaction space from this dataset. The remaining 254 structures were then projected into this constructed reaction space. It is important to note that the C–C bond formations related to all cycloadducts are nearly complete during the descent of the IRCs from TS1 to points with an energy of 5 kcal/mol, as illustrated in Figure 3. Figure 4a shows the two‐dimensional reaction space, where $\Lambda_{i}$ represents the amount of information for each PCo*i*. The two‐dimensional space defined by these two PCos captures 81.6% of the information from the high‐dimensional configuration space, suggesting that the positions of the three TS structures involved in the trispericyclic reaction are accurately represented in this low‐dimensional space.

**FIGURE 4:**
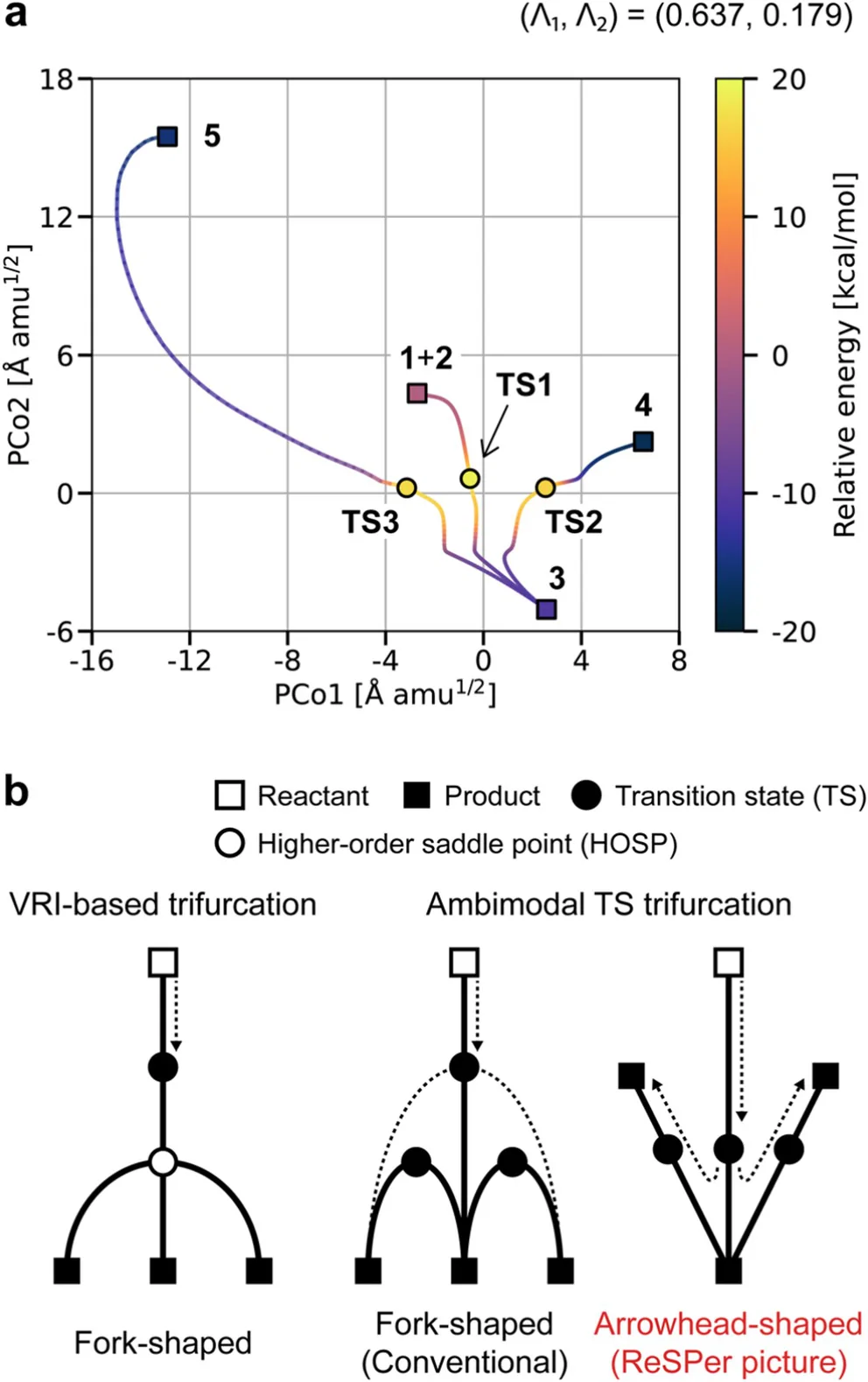
Reduced‐dimensional reaction space involved in the trispericyclic reaction and schematic representations of the potential energy landscape. (a) Two‐dimensional reaction space constructed using ReSPer. Detailed conditions for the ReSPer settings are summarized in Section 3 of the Supporting Information. (b) Schematic illustrations of the potential energy landscape: The conventional fork‐shaped model for post‐transition‐state trifurcation (left and middle) and the proposed arrowhead‐shaped model for the trispericyclic reaction (right).

Investigating the physical meaning of each PCo is crucial for assessing the validity of the reduced‐dimensional reaction space. PCo1 differentiates the three TSs and their corresponding IRCs, showing that bond‐c tends to shorten as PCo1 becomes more positive, while bond‐d tends to shorten as PCo1 becomes more negative. Conversely, PCo2 corresponds to structural changes along IRC1, indicating that bond‐a and bond‐b tend to shorten as PCo2 decreases. Thus, we confirm that the two‐dimensional reaction coordinate determined by ReSPer effectively captures the four C–C bond formations/cleavages using only two PCos.

The reduced‐dimensional reaction space provides valuable insights into the arrangement of reaction paths and the associated potential energy landscape. The reaction‐path picture of a post‐transition‐state trifurcation is often represented by a “fork‐shaped” scheme, where a TS separates the reactants from all possible products within a high‐dimensional potential energy landscape, as depicted in Figure 4b. Consequently, the trispericyclic reaction can be schematically interpreted within this conventional fork‐shaped framework. In contrast, the ReSPer analysis suggests that the two‐dimensional reaction space for the trispericyclic reaction is better represented by an “arrowhead‐shaped” arrangement of reaction paths. In this model, the ambimodal TS is positioned close to other TSs, and the minor products **4** and **5** are structurally closer to the reactant region than the major product **3** in the reduced‐dimensional configurational space. From the perspective of this arrowhead‐shaped scheme, the reaction mechanism can be interpreted as follows: the majority of trajectories passing through the ambimodal TS1 proceed to form cycloadduct **3** along the reaction path, while only a few trajectories diverge dynamically toward other cycloadducts **4** and **5** via IRC‐jumps [42] and energy transfers in directions that allow them to pass over TS2 and TS3.

### 3.4 Approximate Potential Energy Landscape in the Reduced‐Dimensional Reaction Space

Next, we constructed an approximate contour representation of the potential energy around the projected IRC network in the two‐dimensional reaction space (Figure 5). The contour plot was generated from the potential energies of 2415 additional structures projected onto the two‐dimensional reaction space. These structures were obtained by linear interpolation between pairs of molecular structures on neighboring IRCs, namely IRC1 and IRC2 or IRC1 and IRC3. Therefore, the contour map should not be interpreted as a fully relaxed two‐dimensional PES, but rather as an approximate representation of the potential energy landscape around the projected IRC network. Details on the generation of the contour plot are provided in Figure S3 and Section 4 of the Supporting Information. It is important to note that the relative energies shown in the color bar should be regarded as upper bounds to the corresponding relaxed energies because the contour map was constructed from interpolated, nonrelaxed structures.

**FIGURE 5:**
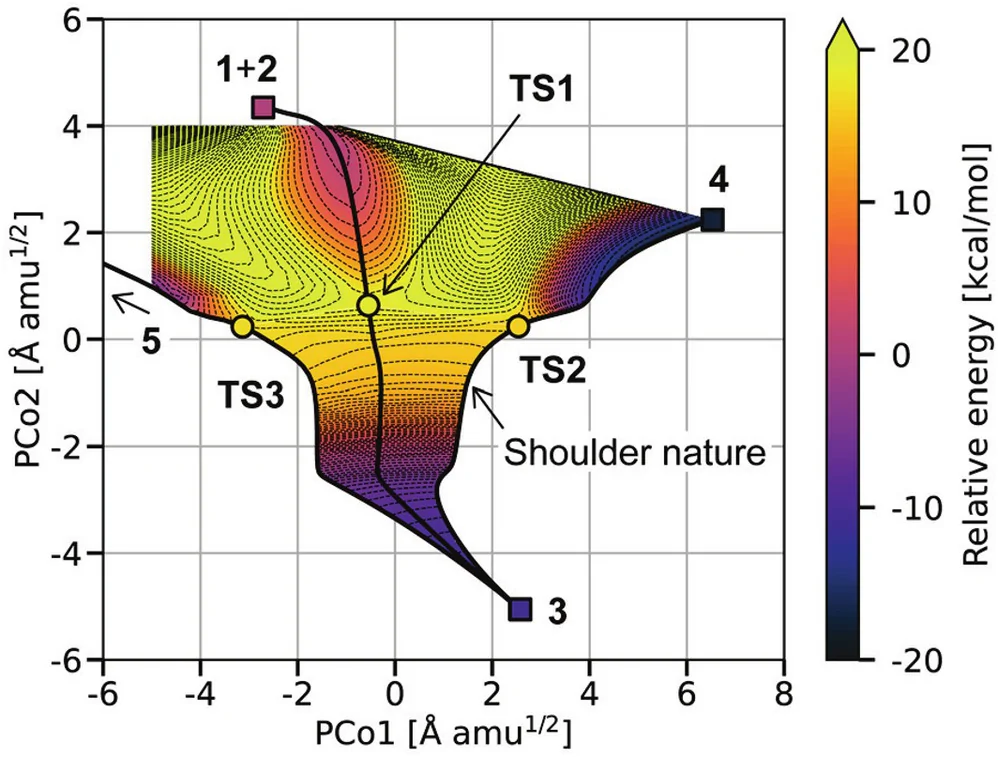
Approximate potential energy landscape around the projected IRC network in the reduced‐dimensional reaction space for the trispericyclic reaction. The contour plot was generated using 2415 structures interpolated between pairs of structures on neighboring IRCs, namely IRC1 and IRC2 or IRC1 and IRC3. Relative energies above 20 kcal/mol are displayed in the same color. Details on the generation of the contour plot are provided in Figure S3 and Section 4 of the Supporting Information.

Figure 5 illustrates the characteristics of the potential energy landscape associated with the trispericyclic reaction. In the contour plot, as the energy descends from the three TSs to the cycloadduct **3**, the energy intervals gradually become sparse within the range of −1 ≦ PCo2 ≦ 0, then suddenly become denser around PCo2 = −1. This pattern indicates that the energy landscape exhibits a shoulder‐like feature within −1 ≦ PCo2 ≦ 0, similar to IRC1. After passing through this shoulder region, the energy drops steeply toward the potential well of **3**. Additionally, in the shoulder region, energy barriers between IRC1 and IRC2 (or IRC3) are lowered due to the close proximity of the three IRCs, as supported by the vibrational analysis along IRC1 shown in Figure 3a. In summary, the reaction mechanisms of the trispericyclic reaction can be explained as follows: Reactive molecules that quickly descend through the shoulder region reach the potential well of **3** and become trapped. However, if the reactive molecules remain in the shoulder region long enough to undergo intramolecular vibrational energy redistributions (IVRs), they may have the opportunity to cross over TS2 or TS3, facilitated by the IRC‐jump process driven by dynamic effects.

The direct reaction channels from reactants **1** + **2** to byproducts **4** and **5** present alternative pathways for the branching reaction. However, the potential energy landscape rises steeply in directions perpendicular to IRC1 on the reactant side, making these pathways less favorable. Therefore, we conclude that the reaction system predominantly proceeds from the reactant region to TS1 rather than directly to **4** and **5**. This mechanism is also supported by the automated reaction path search results discussed in Section 3.1.

### 3.5 Dynamical Branching Processes Projected Onto the Reduced‐Dimensional Reaction Space

To reveal dynamical reaction processes, we projected 120 trajectories, exhibiting the branching ratio of **3**:**4**:**5** = 93:4:3, onto the reduced‐dimensional reaction space. Figure 6a,b shows 112 trajectories (93%) proceeding from TS1 to the potential well of **3**, without and with IRC‐jump, respectively. These trajectories mostly terminated around cycloadduct **3** without crossing over TS2 or TS3, except for one trajectory that recrossed TS2. Despite initiating the AIMD simulations from TS1, 72 trajectories in Figure 6b experienced IRC‐jumps from IRC1 to IRC2 or IRC3 near the IRC1 shoulder region before approaching **3**. This behavior reflects the flatness of the potential energy landscape perpendicular to IRC1 in the shoulder region.

**FIGURE 6:**
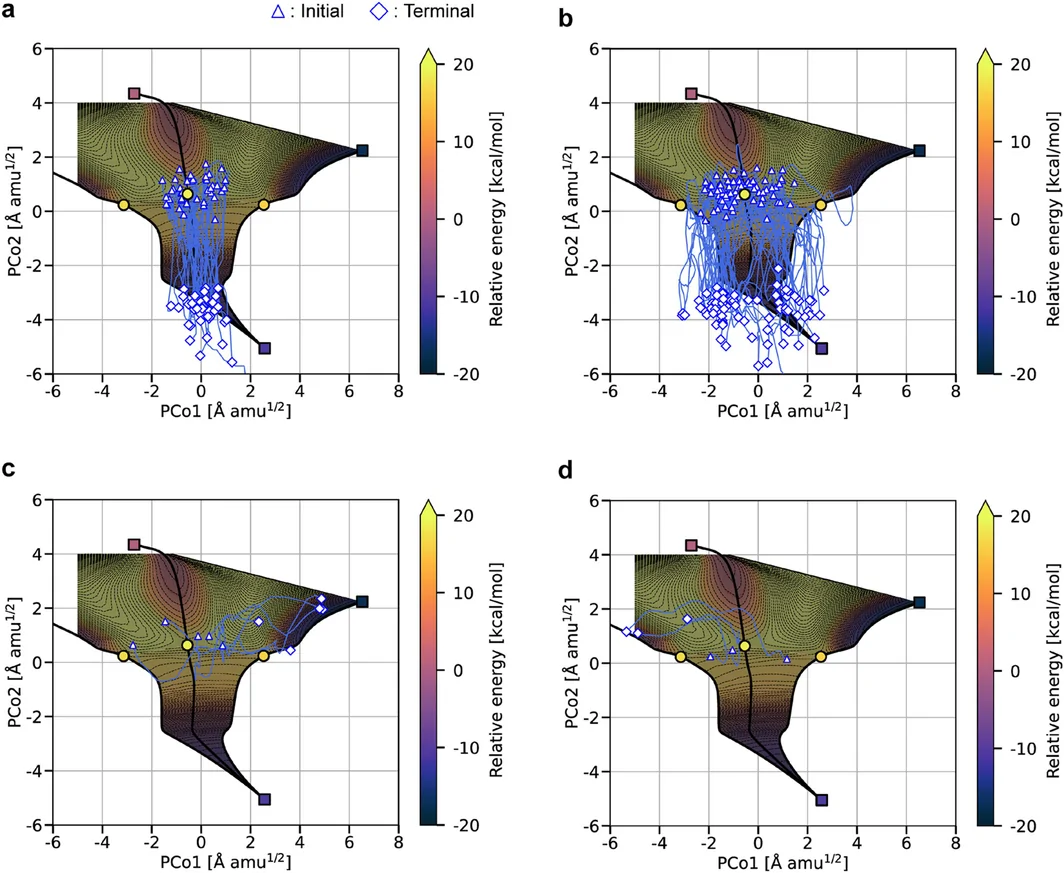
AIMD trajectories projected onto the reduced‐dimensional reaction space. (a) One hundred and twelve trajectories approaching cycloadduct **3** without IRC‐jump processes and (b) with IRC‐jump processes. (c) Five trajectories approaching cycloadduct **4**, and (d) three trajectories approaching cycloadduct **5**. The initial and terminal points are indicated by triangles and diamonds, respectively.

Figure 6c shows five trajectories (4%) that proceeded to cycloadduct **4**, while Figure 6d shows the three trajectories (3%) that proceeded to cycloadduct **5**. Interestingly, these trajectories dynamically diverged toward TS2 or TS3 before approaching **3** in the two‐dimensional potential energy landscape. These dynamical behaviors qualitatively align with the arrowhead‐shaped landscape model; molecules leaving the shoulder region fall into the potential well of **3**, while molecules with sufficient vibrational energy perpendicular to IRC1 may undergo IVR around the shoulder, allowing them to cross over to TS2 or TS3. This interpretation is consistent with a previous report showing that relatively flat regions of the PES can promote nonstatistical dynamics and communication between neighboring TS regions [8].

## 4 Conclusion

In this study, we investigated the dynamical branching mechanism of a trispericyclic reaction using IRC, AIMD, and ReSPer analyses. The IRC analysis from the reactants to cycloadduct **3** revealed that the potential valley perpendicular to the reaction path becomes shallow near the shoulder region, where the potential energy is close to that of the rearrangement TSs connecting **3** to the minor products **4** and **5**. AIMD trajectories initiated from the ambimodal TS gave a branching ratio of **3**:**4**:**5** = 93:4:3 among the reactive trajectories, in quantitative agreement with the previous trajectory study. These results indicate that the shoulder region plays a central role in the dynamical branching process, although the relationship between the static IRC network and the trajectory ensemble remains difficult to clarify from IRC and AIMD analyses alone.

To overcome this difficulty, we applied ReSPer to construct a reduced‐dimensional reaction space without preselecting chemically intuitive internal coordinates. Projection of the IRC paths and AIMD trajectories onto the two‐dimensional reaction space enabled us to visualize the dynamical behavior of trajectories leading to the major product **3**, both with and without IRC‐jump processes, as well as rare trajectories dynamically diverging to the minor products **4** and **5**. The projected trajectories show that branching occurs around the shoulder region, where the potential valley orthogonal to IRC1 becomes shallow, and trajectories with sufficient transverse vibrational motion can approach TS2 or TS3 through IRC‐jump processes.

The ReSPer analysis also revealed that the reaction path network of the trispericyclic reaction is more naturally represented by an arrowhead‐shaped arrangement in the reduced‐dimensional reaction space than by the conventional fork‐shaped schematic picture of post‐transition‐state bifurcation. This representation provides a complementary view of reaction dynamics by preserving mutual structural relationships among the relevant IRCs and transition states. The present study demonstrates that ReSPer can bridge static reaction‐path networks and dynamical trajectory ensembles and suggests that reduced‐dimensional reaction space descriptors may be useful for analyzing and ultimately predicting product branching in complex multichannel reactions.

## Author Contributions

**Takuro Tsutsumi:** conceptualization, data curation, funding acquisition, investigation, methodology, software, visualization, writing – original draft, writing – review and editing. **Akifumi Fujita:** conceptualization, data curation, investigation, methodology, software, visualization. **Yuriko Ono:** conceptualization, methodology, software, visualization, writing – review and editing. **Tetsuya Taketsugu:** conceptualization, funding acquisition, methodology, project administration, resources, supervision, writing – review and editing.

## Funding

This work was supported by Japan Science and Technology Agency (JPMJAX23DE), Japan Society for the Promotion of Science (JP22K14640, JP25H00863), Ministry of Education, Culture, Sports, Science and Technology (JPMXP1122712807).

## Conflicts of Interest

The authors declare no conflicts of interest.

## Supporting information


**Table S1:** The bond lengths (in Å) of the optimized geometries. The corresponding bonds are illustrated in Figure 2a, with the definitions of bond‐a, b, c, and d provided in Figure 2 of the manuscript.
**Figure S1:** The NRO analysis for (a) the IRC connecting **1** + **2** to **3** via TS1, (b) the IRC connecting **3** to **4** via TS2, and (c) the IRC connecting **3** to **5** via TS3. NRO pairs are visualized with an isovalue of 0.05, and electron density changes are depicted with an isovalue of 0.002.
**Figure S2:** The two‐dimensional reaction space determined by (a) all reference structures along the three IRCs and (b) selected reference structures with potential energies above 5 kcal/mol relative to **1** + **2**. The colored lines represent the reference structures, while the black lines represent molecular structures projected onto the preconstructed two‐dimensional reaction space.
**Figure S3:** The projected interpolated structures onto the two‐dimensional reaction space constructed by ReSPer.

## Data Availability

The data supporting the findings of this study are available in the article or Supporting Information. Source data are provided with this paper. Additional raw data are available from the corresponding author on reasonable request.
