# Supplementary material for: Reduced‐Dimensional Reaction‐Space Analysis of a Trispericyclic Reaction by ReSPer
Source: J Comput Chem. 2026 Nov 2;47(29):e70539. doi: 10.1002/jcc.70539 (PMC13728543; doi:10.1002/jcc.70539)
Supplement: Supplementary file 1 [file JCC-47-0-s001.pdf]

Supporting Information for

Reduced-Dimensional Reaction-Space Analysis of a Trispericyclic Reaction by ReSPer

Takuro Tsutsumi[1*], Akifumi Fujita[1], Yuriko Ono[1,2], and Tetsuya Taketsugu[1,3*]

[1] *Department of Chemistry, Faculty of Science, Hokkaido University, Sapporo 060-0810, Japan*

[2] *Institute for Quantum Chemical Exploration, Tokyo 108-0075, Japan*

[3] *Institute for Chemical Reaction Design and Discovery (WPI-ICReDD), Hokkaido University, Sapporo 001-0021, Japan*

*Corresponding author: Takuro Tsutsumi (t.tsutsumi@nagoya-u.jp) and
Tetsuya Taketsugu (take@sci.hokudai.ac.jp)

Table of Contents

1. Structural parameters of minima and transition state structures 3

2. Natural Reaction Orbital Analysis along IRC paths 4

3. Selection of Reference Structures for ReSPer 6

4. Interpolated structures projected onto the two-dimensional reaction space 8

5. Cartesian coordinates for significant structures (in Å) 10

References 19

1. Structural parameters of minima and transition state structures

Table S1. The bond lengths (in Å) of the optimized geometries. The corresponding bonds are illustrated in Figure 2a, with the definitions of bond-a, b, c, and d provided in Figure 2 of the manuscript.

**Bond length / Å**	**bond-a**	**bond-b**	**bond-c**	**bond-d**
**1+2**	3.25	3.25	3.59	3.32
**3**	1.58	1.58	3.43	3.68
**4**	1.56	3.72	1.58	3.39
**5**	1.56	5.26	5.74	1.57
**TS1**	1.99	2.77	3.18	3.00
**TS2**	1.63	2.87	2.60	3.12
**TS3**	1.63	2.98	3.24	2.50

2. Natural Reaction Orbital Analysis along IRC paths

We have recently developed the Natural Reaction Orbital (NRO), which characterizes electron transfer responsive to nuclear coordinate displacement along a reaction coordinate.[1], [2] The pair of occupied and virtual NROs is automatically extracted by applying singular value decomposition to the solution of the coupled-perturbed self-consistent-field (CPSCF) equation for nuclear displacement perturbation. Since the singular values indicate the degree of mixing between occupied and virtual orbitals responsible for structural displacement, scanning the sum of the squared singular values (the Frobenius norm) along a reaction coordinate enables us to capture crucial electron transfers associated with chemical reaction processes.

In this study, we applied NRO analysis to IRC1, IRC2, and IRC3 (originating from TS1, TS2, and TS3) associated with the trispericyclic reaction. All IRCs exhibit two prominent peaks in the Frobenius norm around the respective TSs, indicating a stepwise reaction mechanism in the formation of two C-C bonds. **Figure S1a** shows the potential energy profiles and Frobenius norm along IRC1, with the occupied and virtual NRO pair and corresponding electron density changes (from blue to yellow) shown in the right-hand panels. At peak 1, electronic density changes of the dominant NRO pair reveal electron transfer from the π electron of 6,6- dimethylfulvene (**2**) to the π electron of 8,8-dicyanoheptafulvene (**1**) and the σ electron of bond-a. In contrast, at peak 2, the π electron of **2** transfers to the π electron of **1** with bond-b formation. **Figures S1b and S1c** illustrate the NRO results for IRC2 and IRC3, respectively. The structural changes along IRC2 (IRC3) correspond to bond alternation between bond-b and bond-c (bond-d). At peak 1 in both IRC2 and IRC3, electron density changes of the dominant NRO pair show electron transfers from the σ electron associated with bond-b to the π electron of **1**. Subsequently, at peak 2 near TS2, the π electron of **1** shifts to the σ electron of bond-c, while near TS3, electron density concentrates at the center of bond-d. This indicates that bond-a likely strengthens along IRC3 to facilitate bond-d

formation, influenced by steric hindrance from the bulky regions of **1** and **2**. Electron density changes in the secondary dominant NRO pair show the gathering of the σ electron at bond-d.

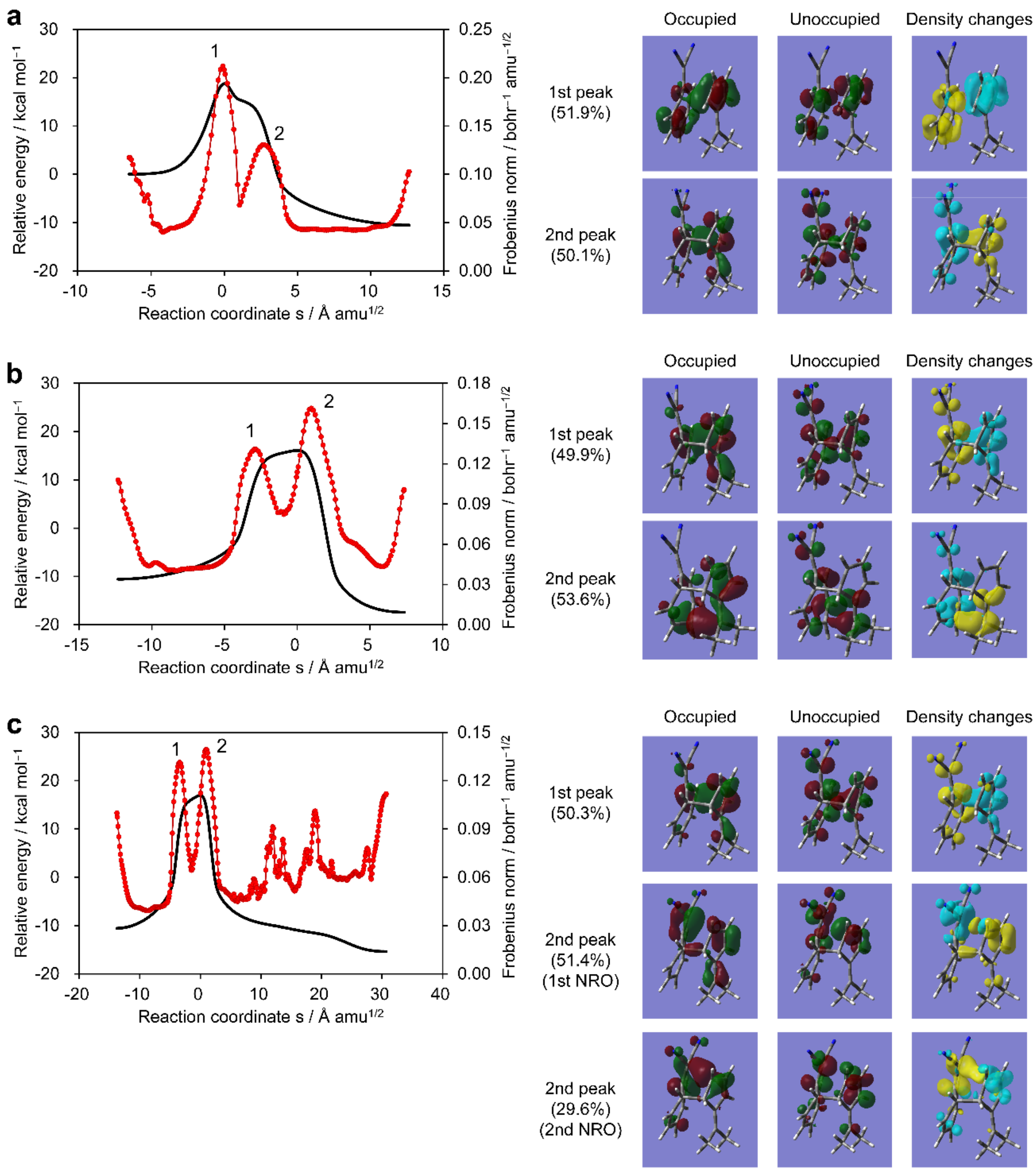

Figure S1. The NRO analysis for (a) the IRC connecting **1**+**2** to **3** *via* TS1, (b) the IRC connecting **3** to **4** *via* TS2, and (c) the IRC connecting **3** to **5** *via* TS3. NRO pairs are visualized with an isovalue of 0.05, and electron density changes are depicted with an isovalue of 0.002.

3. Selection of Reference Structures for ReSPer

ReSPer extracts the principal coordinates (PCos) to maximize the structural dissimilarity distribution among the reference structures. Thus, the selection of reference structures influences the interpretation of PCos, which define the low-dimensional reaction space. **Figure S2a** illustrates the two-dimensional reaction space determined from all reference structures along the three IRCs, totaling 325 reference structures. For simplicity, the forward and backward directions of the three IRCs were thinned to approximately 50 molecular structures. **Figure S2a** indicates that the region with negative PCo1 primarily reflects structural changes along IRC3 from TS3 to **5**, due to the relatively extended length of the partial IRC starting from TS3 to **5** compared to other pathways. To accurately capture the potential energy landscape around the ambimodal TS (TS1) region, we selected 71 reference structures with relative energies above 5 kcal/mol relative to **1+2**. It is worth noting that the formation and cleavage of the relevant C-C bonds were nearly completed within this set of selected reference structures. **Figure S2b** shows the two-dimensional reaction space determined by these 71 selected reference structures, with 254 molecular structures of potential energy below 5 kcal/mol projected using the out-of-sample extension technique.[3]

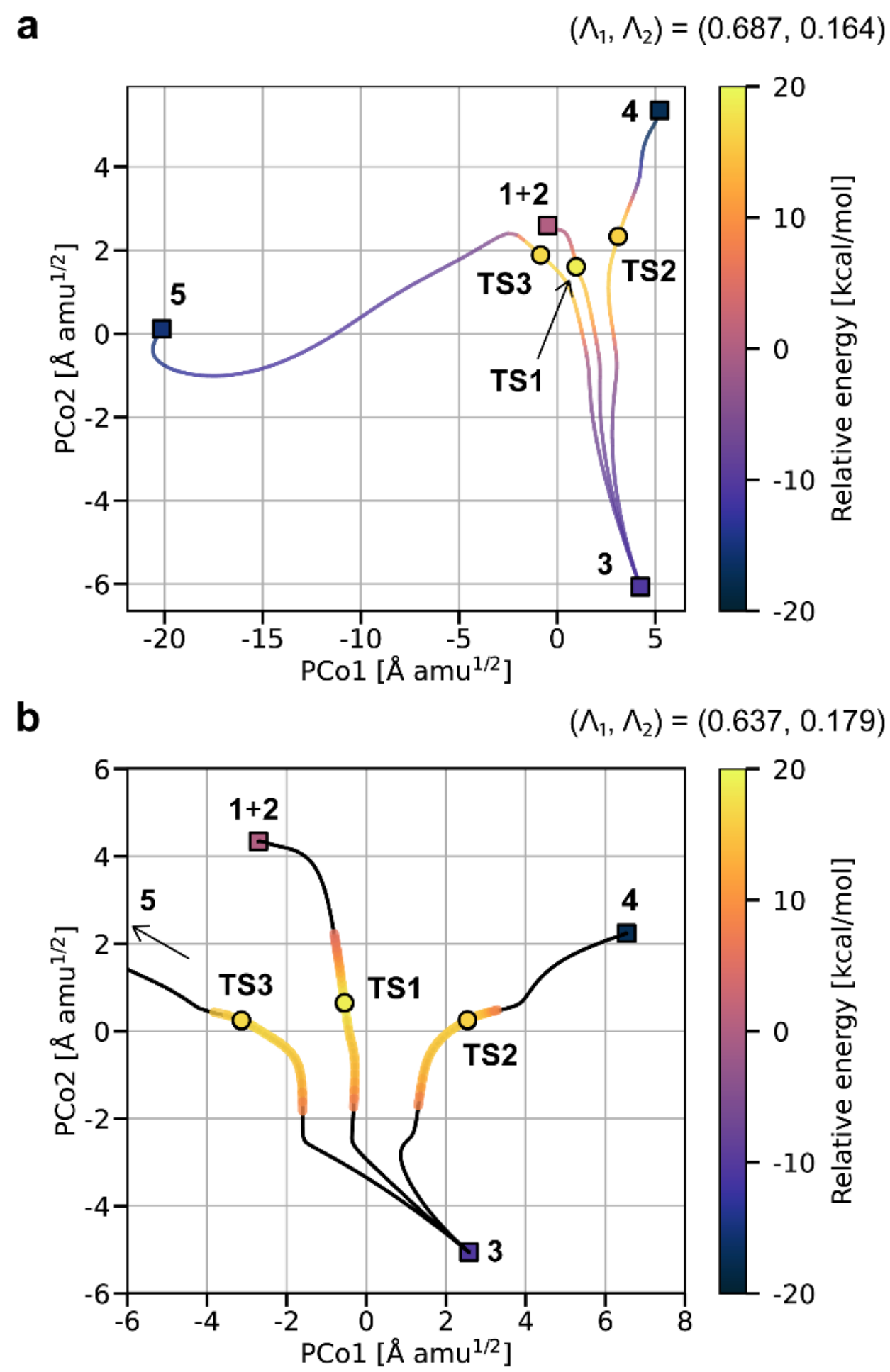

Figure S2. The two-dimensional reaction space determined by (a) all reference structures along the three IRCs and (b) selected reference structures with potential energies above 5 kcal/mol relative to **1+2**. The colored lines represent the reference structures, while the black lines represent molecular structures projected onto the preconstructed two-dimensional reaction space.

4. Interpolated structures projected onto the two-dimensional reaction space

The potential energy contours in the two-dimensional reaction space constructed by ReSPer (**Figure 5** in the manuscript) were generated by linear interpolation between neighboring IRCs. The interpolation path was defined by linearly interpolating between two molecular structures selected from neighboring IRCs (e.g., IRC1 and IRC2, IRC1 and IRC3). Each IRC was thinned so that each forward and backward path contained approximately 50 structures. For instance, in the case of IRC1 and IRC2, interpolation points were chosen as follows: TS1 and TS2; the first step of IRC1 and the first step of IRC2; and so forth. By dividing each interpolation path into 20 grid points, we obtained 2,415 interpolation structures in the $3N$-dimensional Cartesian coordinate space. These interpolated structures were projected onto the two-dimensional reaction space constructed by ReSPer, and energy contours were generated by spline interpolation of the potential energy. The resulting contour map should therefore be interpreted not as a fully relaxed two-dimensional potential energy surface, but rather as an approximate representation of the potential energy around the projected IRC network. In **Figure 5**, the contours were truncated within the range of PCo1 ≥ -5 and PCo2 ≤ 4 due to technical limitations in rendering.

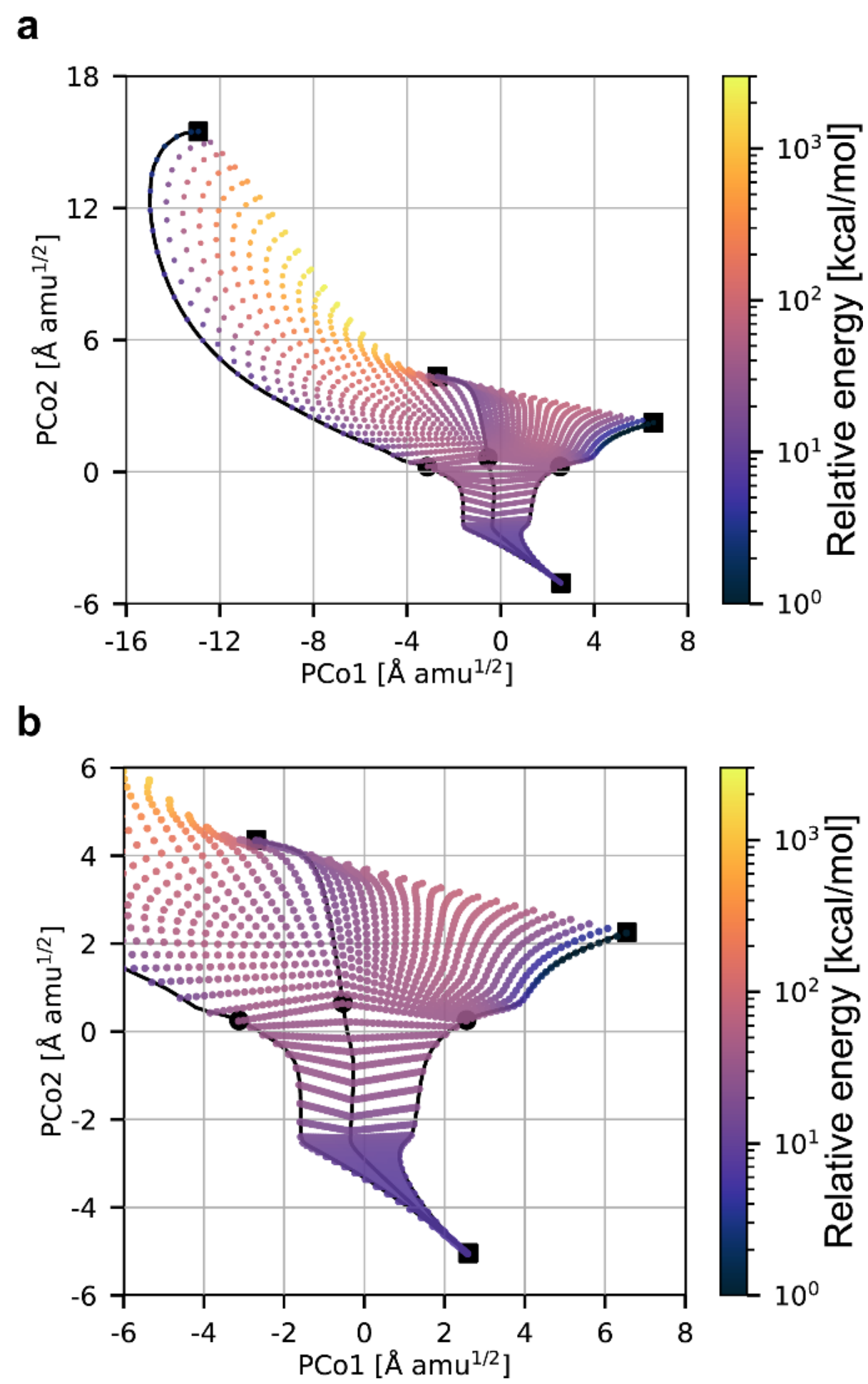

Figure S3. The projected interpolated structures onto the two-dimensional reaction space constructed by ReSPer.

5. Cartesian coordinates for significant structures (in Å)

1+2(Reactant) E= -804.652644025858 Hartree

C	1.092769753046	1.014342537282	0.155887450780
C	0.453580714270	1.067956594616	1.451974438898
C	0.450049801199	1.457507348638	-1.061380833343
C	-0.735637005878	1.609533344733	1.822139311681
C	-0.740049486028	2.085061337746	-1.246917787061
C	-1.731926864061	2.275132025855	1.042844003612
C	-1.733888247624	2.483048417790	-0.299899743434
H	-0.969847644401	1.523687222514	2.880604353324
H	-2.587607846631	2.647684264800	1.600602577906
H	-2.590974219978	3.006537760288	-0.716403322643
H	-0.977365383093	2.323417619168	-2.281043757900
C	-0.431429202613	-1.645303099130	-1.438950468920
H	1.023267400023	1.267388340053	-1.964098968518
C	-0.432974986131	-2.004223169846	0.875846740807
H	1.028987441755	0.613447966970	2.253175996047
C	-1.262585546002	-1.499949166169	-0.231730954235
C	0.750660617364	-2.415287701912	0.367998574128
H	-0.729502995003	-2.048618727161	1.915275407882
C	0.751643052542	-2.190995303178	-1.077781730637
H	-0.726561609695	-1.372327942319	-2.443293079762
H	1.579857506151	-2.828587576062	0.929830998195
H	1.581637524514	-2.414930042368	-1.737320679897
C	-2.521778036573	-1.015915968517	-0.157468841500
C	-3.290602850119	-0.547175648048	-1.360852278263
C	-3.292217408429	-0.933991533323	1.130500557302
H	-2.695604518252	-1.194189869505	2.005599042921

H	-4.158499617782	-1.607218599194	1.094659102584
H	-3.687664475156	0.079379797306	1.271472025981
H	-2.697950806223	-0.546463533259	-2.276366446792
H	-3.670181606851	0.468594186474	-1.194901560142
H	-4.167189771994	-1.188107149385	-1.521174348618
C	2.367100891987	0.472123274630	0.069996310194
C	3.046330832498	-0.036383023326	1.215517872999
N	3.578198092280	-0.451750784923	2.161011089852
C	3.043023502171	0.335359870962	-1.177656406916
N	3.572407392923	0.226721984406	-2.205915820225

3 **E= -804.669467128201 Hartree**

C	1.102353194690	0.177804546688	0.026389957614
C	0.337636479956	-0.194988384779	1.273016577486
C	0.335291716541	0.203845478766	-1.273172555528
C	-0.464346329397	0.977549928958	1.775071138501
C	-0.467295012609	1.473392502637	-1.391420930783
C	-1.020505632273	1.960270358579	1.050487217295
C	-1.022004326532	2.187302080740	-0.400127069250
H	-0.607396646389	1.001019270122	2.852871426732
H	-1.569137811674	2.713732333989	1.611605400224
H	-1.571724842509	3.075886106819	-0.703134141792
H	-0.612462425486	1.824964335800	-2.410249401431
C	-0.570021413229	-1.088539737065	-1.359512841041
H	1.045922698706	0.172412366617	-2.105288601938
C	-0.567756493521	-1.452255083647	0.961850005897
H	1.049685704386	-0.478949261116	2.054510805542
C	-1.499168885813	-1.067559160215	-0.166093164406

C	0.292852889240	-2.501556367284	0.283186902856
H	-1.047744530377	-1.783498604035	1.884312107157
C	0.291637574643	-2.294775924422	-1.035915791619
H	-1.051364148745	-1.122340582669	-2.338359387662
H	0.883874203789	-3.236222750698	0.819080608363
H	0.881482483746	-2.830138265916	-1.771912425291
C	-2.791404848289	-0.740458564529	-0.113533493159
C	-3.584037772783	-0.324247518686	-1.324904165269
C	-3.581199687548	-0.714427119857	1.168806161184
H	-3.006508615895	-1.041729034293	2.037185730293
H	-4.471499217468	-1.350619961413	1.088568032931
H	-3.933338508201	0.305048393262	1.370479921820
H	-3.011701139793	-0.371554165802	-2.253165253495
H	-3.935400310587	0.708339732680	-1.205271453629
H	-4.474914177352	-0.954235304633	-1.440119948072
C	2.415525630494	0.501346597558	0.075792456004
C	3.150488370759	0.500452951792	1.309717595211
N	3.731192765810	0.490519961331	2.312905182078
C	3.148117460486	0.877791508808	-1.100818742333
N	3.726883176346	1.175038398335	-2.060196160746

4 **E= -804.680405327494 Hartree**

C	1.132777884950	0.214774861627	0.697681668455
C	0.417811010404	-1.122659728831	0.677366314854
C	0.440517178901	1.469207325403	0.971844312025
C	-0.504613906844	-1.326501275252	1.858096548671
C	-0.866366809045	1.654974403597	1.216664376633
C	-1.575371794060	-0.576541382180	2.090833193612

C	-1.961644792696	0.623027343891	1.253301990381
H	-0.279098387832	-2.166026524051	2.510224737730
H	-2.219239191421	-0.814312915706	2.933732663585
H	-2.830394425836	1.088352605612	1.731580731012
H	-1.180748059578	2.683830431155	1.377026403457
C	-0.915068731116	0.249848979053	-2.270440926304
H	1.071631976563	2.353593315139	0.968949370900
C	-0.337865634765	-1.331453468524	-0.674291827271
H	1.179420793900	-1.905243283684	0.715789768931
C	-1.288483351006	-0.210464639472	-1.060218979335
C	0.593245788902	-1.420340527924	-1.852173065940
H	-0.890395352789	-2.275721767806	-0.567624889469
C	0.244832124668	-0.498705699714	-2.761754260666
H	-1.389144732190	1.053481980175	-2.821809354167
H	1.415946733615	-2.123149746037	-1.908293869096
H	0.726281370760	-0.323523038188	-3.716964028065
C	-2.444350394742	0.258188410884	-0.208705553221
C	-3.089829045653	1.509950682001	-0.820648955811
C	-3.513873612518	-0.845807734227	-0.114067968746
H	-3.108982388186	-1.768395682877	0.310637392424
H	-3.907894293891	-1.071743553714	-1.109982640540
H	-4.348334278973	-0.518728597862	0.518243864744
H	-2.367134338910	2.324600462979	-0.933403372489
H	-3.912242909755	1.863067166530	-0.188662535062
H	-3.504944088414	1.286557179550	-1.808611441711
C	2.471270240803	0.253083946232	0.419201204917
C	3.227146710775	-0.929879475540	0.129343661572
N	3.831736987906	-1.891822467790	-0.104748904000

C	3.214064842447	1.479306830776	0.377825104361
N	3.810544911574	2.473268247615	0.346629014679

5 **E= -804.677132396943 Hartree**

C	0.982537485285	-0.989597627255	0.566230618239
C	-0.401016919266	-0.847426609575	-0.010660527471
C	1.351709640451	-1.955633244735	1.423154815099
C	-1.294675274310	-0.475682623737	1.145900927831
C	0.428154979834	-2.878993235133	2.040315310266
C	-1.534311156755	-1.336143800747	2.152147720797
C	-0.863360955466	-2.601520164242	2.359076987671
H	-1.769954975189	0.499861465644	1.147888994329
H	-2.273644957900	-1.059232894503	2.901113443546
H	-1.401686431352	-3.345964922773	2.942199706197
H	0.844478508996	-3.822304169614	2.385705260854
C	-0.781090346188	2.428868969984	-0.537906625447
H	2.401276047011	-2.048261774591	1.698009565338
C	-0.281242829989	0.184019708582	-1.169366569098
H	-0.732237042142	-1.819795038096	-0.400474952466
C	-1.367636983709	1.250275743870	-1.189751669904
C	1.025183018315	0.979776098314	-0.909062432072
H	-0.217978463266	-0.358136892062	-2.115884038393
C	0.527775167200	2.271657971012	-0.310798578353
H	-1.341757034720	3.315802078428	-0.265591364359
H	1.567101928641	1.197884134618	-1.836179052291
H	1.183898853260	2.996758329493	0.157420617229
C	-2.615401014974	1.100512489232	-1.661615420307
C	-3.645773875494	2.198032881036	-1.641242379603

C	-3.110594891902	-0.198788834109	-2.238581056935
H	-2.341167836126	-0.972864316925	-2.280022370016
H	-3.501174627682	-0.050898024810	-3.253346137902
H	-3.940806572493	-0.587760652688	-1.634758214077
H	-3.270479709350	3.138629037065	-1.233817983438
H	-4.518225393446	1.894680702662	-1.048389804505
H	-4.010284420643	2.395908500327	-2.657220381244
C	1.933977127585	0.067039228246	-0.012727272847
C	2.939153650328	-0.606243776226	-0.858929800004
N	3.696377660772	-1.146731645337	-1.546918438322
C	2.630638970866	0.827600766669	1.037934552930
N	3.157425792244	1.437914640366	1.867678607501

TS1 **E= -804.622752813549 Hartree**

C	1.075586000000	0.765709000000	0.074467000000
C	0.339136000000	0.470167000000	1.312144000000
C	0.425979000000	1.197454000000	-1.113182000000
C	-0.721210000000	1.304534000000	1.799601000000
C	-0.805407000000	1.838679000000	-1.261613000000
C	-1.639647000000	2.036035000000	1.093005000000
C	-1.729651000000	2.209972000000	-0.301954000000
H	-0.847866000000	1.285808000000	2.880029000000
H	-2.406938000000	2.534756000000	1.681730000000
H	-2.595277000000	2.763063000000	-0.658927000000
H	-1.053015000000	2.109595000000	-2.285719000000
C	-0.452025000000	-1.420912000000	-1.347058000000
H	1.037098000000	1.156235000000	-2.010553000000
C	-0.384639000000	-1.351826000000	0.978926000000

H	1.001316000000	0.160762000000	2.116460000000
C	-1.285565000000	-1.151543000000	-0.192942000000
C	0.741167000000	-2.055749000000	0.478766000000
H	-0.767819000000	-1.545167000000	1.974772000000
C	0.713016000000	-2.040622000000	-0.922164000000
H	-0.740979000000	-1.281163000000	-2.379797000000
H	1.543069000000	-2.453265000000	1.088655000000
H	1.504188000000	-2.402044000000	-1.567331000000
C	-2.603089000000	-0.840945000000	-0.178756000000
C	-3.418378000000	-0.713512000000	-1.434630000000
C	-3.393524000000	-0.719551000000	1.092832000000
H	-2.766393000000	-0.630023000000	1.980624000000
H	-4.034055000000	-1.603576000000	1.213430000000
H	-4.055891000000	0.151743000000	1.053521000000
H	-2.815721000000	-0.723723000000	-2.343507000000
H	-3.991953000000	0.219759000000	-1.415529000000
H	-4.145682000000	-1.533224000000	-1.497813000000
C	2.420526000000	0.390712000000	0.029409000000
C	3.109260000000	-0.098193000000	1.175853000000
N	3.647142000000	-0.504001000000	2.124032000000
C	3.168480000000	0.463443000000	-1.180911000000
N	3.743474000000	0.511039000000	-2.189826000000

TS2 **E= -804.626992913420 Hartree**

C	1.077905076610	0.202250412982	0.627919429116
C	0.342686462065	-1.117510698218	0.657363886536
C	0.404856151354	1.456384421377	0.686795286211
C	-0.569104802337	-1.300253468794	1.824919555035

C	-0.867692391650	1.734158866104	1.172669870579
C	-1.529666402407	-0.451112843572	2.234622075192
C	-1.800235430461	0.866985182930	1.722243985000
H	-0.478383708625	-2.254262362521	2.338494482108
H	-2.191146807926	-0.792659803207	3.028889008425
H	-2.732158852614	1.317657335771	2.056674608863
H	-1.183313541124	2.770564787089	1.070866618972
C	-0.587653606208	0.670674807895	-1.887887531683
H	1.006433796868	2.318632361227	0.412614212192
C	-0.392124538034	-1.363437687246	-0.779402113879
H	1.084458296267	-1.916867014680	0.702316585121
C	-1.323712743248	-0.204424775202	-1.076446200207
C	0.666099015260	-1.221219731577	-1.812992640337
H	-0.851587920672	-2.351992142572	-0.723061974414
C	0.565424245514	0.002072600823	-2.398647927142
H	-0.908499866818	1.655370754071	-2.203840979442
H	1.466291300241	-1.937772786822	-1.954150994400
H	1.259741826784	0.426512638307	-3.113191145913
C	-2.600699760738	-0.000416115685	-0.589114256474
C	-3.385481802720	1.222313022398	-0.988273680830
C	-3.428056470541	-1.137906710072	-0.057557237508
H	-2.825212551281	-1.934710822677	0.378983013559
H	-4.008963142570	-1.565417136257	-0.887284568629
H	-4.144355757201	-0.793263964042	0.694101118423
H	-2.758248327004	2.110373556494	-1.087696425414
H	-4.163836968346	1.436831766096	-0.250423764854
H	-3.887036347883	1.050209086035	-1.950183717522
C	2.437569905071	0.161938032136	0.348487659307

C	3.153829748803	-1.061558414938	0.186293171614
N	3.723739821381	-2.065840425337	0.051081724994
C	3.201601778060	1.357746855408	0.188903248243
N	3.798830316131	2.343378416275	0.044684619158

TS3 **E= -804.625538021863 Hartree**

C	1.003366030881	-0.699119747458	0.531944405970
C	0.240687003127	-1.171750654252	-0.674470871346
C	0.423521410140	-0.297351377921	1.716265942971
C	-0.842773970971	-2.172244220264	-0.458391274799
C	-0.891707931093	-0.562178560787	2.210533378574
C	-1.741414510107	-2.234374174185	0.542545697360
C	-1.844658490835	-1.387756040347	1.704455191539
H	-0.925488575730	-2.924387480035	-1.240511527361
H	-2.490951360117	-3.021072609192	0.474810916210
H	-2.760017828866	-1.492165872087	2.282757186079
H	-1.130647250965	-0.060376045782	3.145620266464
C	-0.431543484299	2.020416806361	0.044924785945
H	1.078670573356	0.216227444918	2.416022596485
C	-0.178175555811	0.206086242142	-1.436468005278
H	0.951591063754	-1.625285328629	-1.370235538534
C	-1.193011510725	0.988957740955	-0.620151377338
C	1.018032410866	1.102181628755	-1.431770698482
H	-0.500282496525	-0.066238112249	-2.444308907185
C	0.807917592132	2.162927637380	-0.551149195817
H	-0.818533281123	2.682856189021	0.809219678040
H	1.838232271102	1.010380150486	-2.132368690272
H	1.540473865014	2.921395621979	-0.305195521306

C	-2.530856246324	0.809720898653	-0.570060628461
C	-3.432131972797	1.634121514144	0.307836178266
C	-3.260445342035	-0.155031055071	-1.461742196305
H	-2.595236300041	-0.780501030096	-2.057652306034
H	-3.915421763698	0.402797669416	-2.144557484505
H	-3.903784471091	-0.817281903686	-0.871740848437
H	-2.893360886878	2.268581443829	1.012856193057
H	-4.089073605774	0.974217312639	0.886037069682
H	-4.081866201285	2.274915120206	-0.302000292751
C	2.322341118151	-0.274851187802	0.192908780470
C	3.076757898251	-0.890428789381	-0.848687833889
N	3.671170743075	-1.371249009308	-1.725158056037
C	3.041410357889	0.612263593557	1.046536046414
N	3.570058699350	1.387284184093	1.732398940609

References

[1] S. Ebisawa, M. Hasebe, T. Tsutsumi, T. Tsuneda, T. Taketsugu, *Physical Chemistry Chemical Physics*, **2022**, DOI:10.1039/D1CP04491E.

[2] S. Ebisawa, T. Tsutsumi, T. Taketsugu, *J. Chem. Phys.*, **2022**, DOI:10.1063/5.0098230.

[3] T. Tsutsumi, Y. Ono, Z. Arai, T. Taketsugu, *J. Chem. Theory Comput.*, **2020**, DOI:10.1021/acs.jctc.0c00018.
